# An Esophagogastroduodenal Crohn's-Like Disease in a Long-Standing Pan-Ulcerative Colitis Patient

**DOI:** 10.1155/2014/464139

**Published:** 2014-09-10

**Authors:** Christopher Moore, Shriram Jakate, Ali Keshavarzian

**Affiliations:** ^1^Department of Internal Medicine, Division of Digestive Diseases and Nutrition, Rush University Medical Center, Chicago, IL 60612, USA; ^2^Department of Pathology, Rush University Medical Center, Chicago, IL 60612, USA

## Abstract

Inflammatory bowel disease (IBD) comprises the principal subtypes Crohn's disease (CD) and ulcerative colitis (UC), with a fraction remaining as IBD unclassified (IBDU). Given the complexity of IBD manifestations in a patient over time and our increasing understanding of IBD biology, a modification in subtype diagnosis can also occur. Herein is a case of a 27-year-old female with well-controlled and long-standing pan-UC, who developed Crohn's-like esophagogastroduodenitis. The difficulty in classifying IBD into a single traditional subtype, and the debated presentation of a coexistent IBD will be discussed.

## 1. Introduction

Inflammatory bowel disease (IBD) comprises two main subtypes, Crohn's disease (CD) and ulcerative colitis (UC). In terms of anatomic location, CD has a predilection for the ileocecum but anywhere along the gastrointestinal tract (GI) is possible [1]. UC traditionally arises from the rectum and can extend proximally to encompass the entire colon, with effects even upon the ileum, that is, “backwash ileitis” [2]. Associative disease in the upper GI tract is not well characterized and is considered atypical in UC. Despite the addition of extensive clinical and histologic testing, 10% of patients evade a single IBD subtype diagnosis [3] and are designated as IBD unclassified (IBDU). The prior designation of “indeterminate colitis” is now used only after pathological assessment of a resected colon. Further data or expert opinion may direct a resubtyping of the initial diagnosis, with significant therapeutic implications [4].

Herein is a case report of a 27-year-old female with well-controlled and long-standing pan-UC on mesalamine who manifested CD-like features in the esophagus, stomach, and proximal duodenum. The implications upon the validity of her original UC diagnosis, the notion of atypical UC with upper GI involvement, and the potential for the much disputed coexistent CD are all discussed.

## 2. Case Report

A 27-year-old African American healthy female, without IBD family history, was initially evaluated for diffuse abdominal pain and bloody bowel movements in 2008. Her colonoscopy revealed loss of vascular pattern, mucosal granularity, friability, and salt-and-pepper ulceration diffusely involving the rectum and entire colon. Her ileal mucosa was grossly normal. Histological examination revealed crypt distortion, neutrophilic crypt abscesses, and mucin depletion of the colon and rectum, with normal ileal mucosa. Given these findings she was diagnosed with pan-UC. This flare presentation was successfully treated with an oral prednisone pulse with taper, and with maintenance mesalamine 2.4 grams twice daily.

Until late December 2010 she was in clinical remission when she displayed symptoms of a typical UC flare and new normocytic anemia (albumin 2.9 g/dL and hemoglobin 11.8 g/dL). She underwent colonoscopy with biopsies, redemonstrating severe pan-UC and a normal ileum. Furthermore, for her new anemia, she underwent same-day esophagogastroduodenoscopy (EGD) with biopsies, and it demonstrated unremarkable esophagus, stomach, and duodenum. She again responded to an oral prednisone pulse dose with long taper and was able to continue on the same mesalamine dosage.

**Figure 1:**
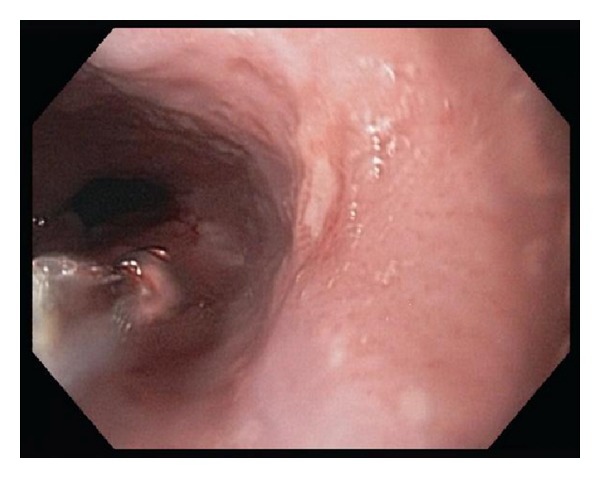
EGD in 2012 which displayed severe prominent ulceration with edematous friable mucosa in the distal esophagus.

**Figure 2:**
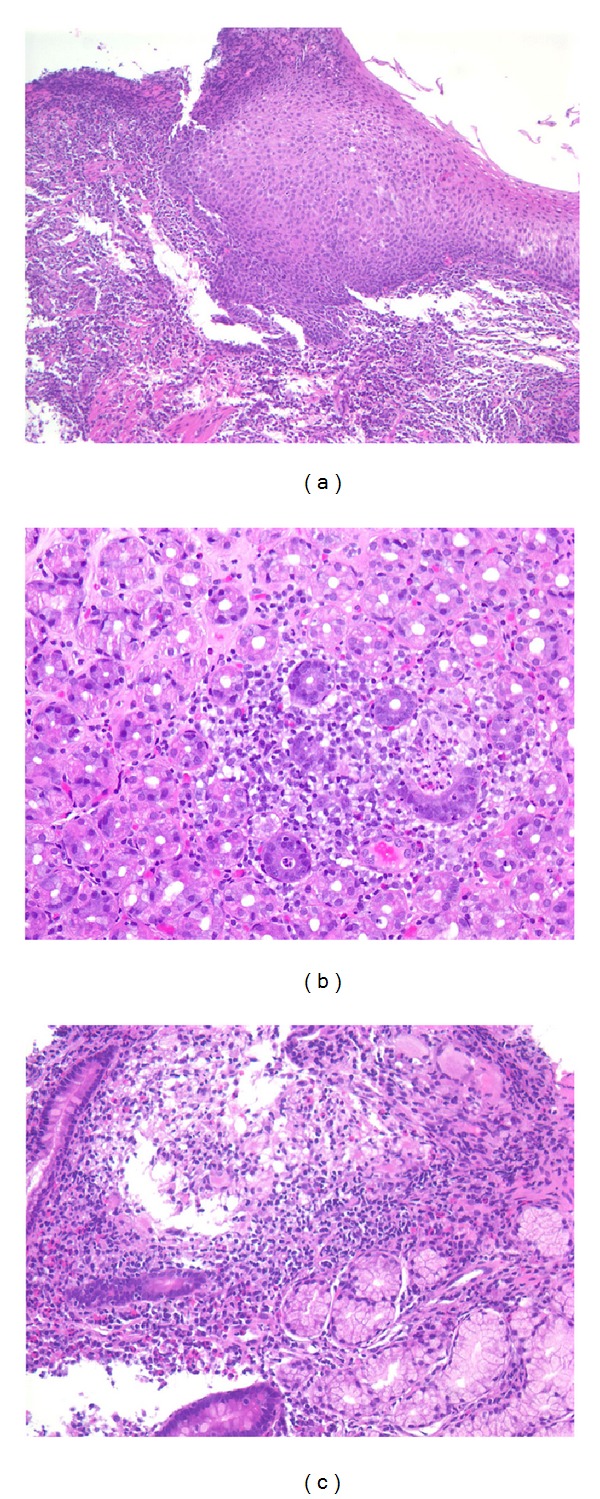
(a) Esophageal biopsy from EGD 2012 showing focal ulceration and patchy acute and chronic esophagitis suggestive of Crohn's disease (routine hematoxylin and eosin staining, magnification ×100). (b) Gastric biopsy from EGD 2012 showing patchy and deep “focally enhancing” gastritis with cryptitis suggestive of Crohn's disease (routine hematoxylin and eosin staining, magnification ×200). (c) Duodenal biopsy from EGD 2012 showing patchy chronic duodenitis with cryptitis and lymphohistiocytic aggregate (routine hematoxylin and eosin staining, magnification ×200).

In early September 2012 she noted increasing solid food dysphagia with associated epigastric pain, nausea, and vomiting. However, she did not manifest any lower GI symptoms as previously experienced during her UC flares. She denied any new medications, foods, behavioral changes, or stressors, and despite a two-week course of proton-pump inhibitor (PPI), her symptoms persisted. Thus, in late September 2012, an EGD was performed and displayed severe prominent ulceration with edematous friable mucosa in the distal esophagus without stricture or fistula, a normal appearing* z*-line, and no hiatal hernia (Figure 1). The stomach displayed a normal appearing fundus and proximal body, whereas the distal body and antrum displayed only a patchy granular pattern. The duodenum was grossly normal in appearance. Histology of esophageal mucosa displayed lymphohistiocytic aggregates with fissuring ulceration (Figure 2(a)). Histology of the gastric mucosa also showed changes typical for focal enhancing gastritis with cryptitis, lymphohistiocytic aggregates, and eosinophilia and was without evidence of* Helicobacter pylori* (Figure 2(b)). Histology of the duodenal mucosa displayed cryptitis and lymphohistiocytic aggregates and eosinophilia (Figure 2(c)). These endoscopic and histological changes were suggestive of a CD-like presentation of the upper GI tract.

**Figure 3:**
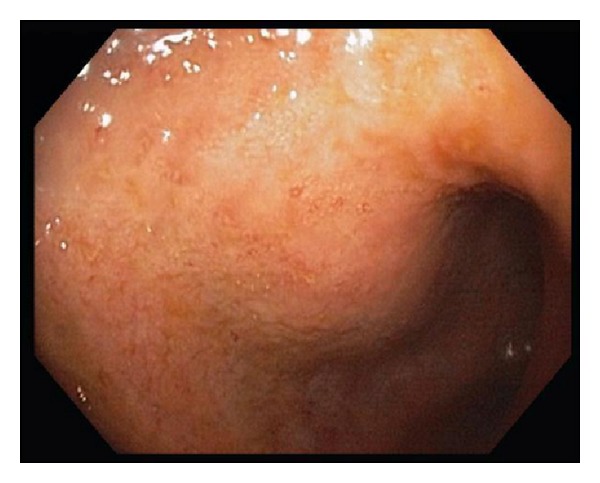
Colonic mucosa on colonoscopy 2013 showing mild active pan-colitis with patchy granularity, altered vascular pattern, and ulcerations.

**Figure 4:**
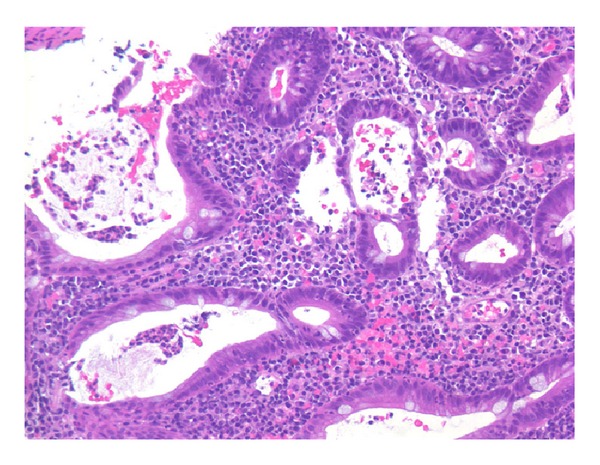
Colonic biopsy on colonoscopy 2013 showing diffuse ulcerative colitis with increased inflammatory cells in the lamina propria, distorted and muco-depleted crypts, cryptitis, and crypt abscesses (routine hematoxylin and eosin staining, magnification ×200).

The patient was started on budesonide 0.25 mg/2 mL inhaled solution to be “swallowed” three times per day and budesonide 3 mg capsule three times per day (capsule was opened and powder was consumed with honey). In the intervening months she reported some improvement in her symptoms, with gradual taper of the budesonide and continuance of her PPI therapy. Given these findings suggestive of a CD-like process in a background of long-standing UC, repeat bidirectional endoscopy was performed four months later in January 2013. The colonoscopy revealed mildly active pan-colitis with patchy granularity, altered vascular pattern, some friability, and a few ulcerations (Figure 3). The colonic biopsies displayed mildly active disease: increased inflammatory cells in the lamina propria, distorted and muco-depleted crypts, cryptitis, and crypt abscesses, without any granulomas (Figure 4). The ileal biopsies were normal. The EGD redemonstrated mucosal diffuse granularity but without friability in the esophagus, stomach (also with small ulceration), and duodenal bulb. The second and third duodenal portions were grossly normal. On histology there were cryptitis, lymphohistiocytic infiltrate, and eosinophilia of the esophagus, stomach, and duodenal bulb suggestive of a CD-like process, but without granulomas.* H. pylori* and dysplasia were negative. The patient continued on her standard mesalamine dosage; she had apparently self-tapered this medication in recent months.

Given these persistent findings, an IBD serologic panel was obtained in February 2013 with the finding “pattern consistent with IBD, inconclusive for CD versus UC” (see Table 1). Overall, she has performed well and currently denies any GI symptoms and continues on her mesalamine and PPI therapy.

## 3. Discussion

IBD involves an interplay of genetic and environmental factors, for which we have incomplete but progressive understanding [1, 2]. In most cases IBD can be dichotomized into CD or UC, although approximately 10% will remain IBDU. Furthermore, approximately 10% of IBD patients will be resubtyped, with implications upon medical and surgical treatments [3, 4].

This case presentation, in which a 27-year-old female with long-standing and well-controlled pan-UC presented with findings suggestive of a CD-like process in the esophagus, stomach, and proximal duodenum, is interesting for a number of reasons. Her initial UC diagnosis was quite strong given her clinical history, her endoscopic and histopathologic findings, and her response to mesalamine. It is noted however that this treatment can elicit a response in CD colitis as well.

Given her new upper GI symptoms in 2012, endoscopy was performed and yielded the findings of cryptitis and lymphohistiocytic infiltrate, which can be a precursor to granuloma formation;* H. pylori* biopsy was negative. To potentially discriminate her diagnosis further, an IBD serologic panel was consistent with IBD but inconclusive for CD versus UC. It is acknowledged that in this patient we did not find granulomas or signs of penetrating disease, which would have been overtly suggestive for a diagnosis of CD, though their absence does not rule it out. Furthermore, the IBD serologic panel can be limited in discriminatory power.

The Montreal Classification of IBD [5] allows for an upper GI manifestation in CD, but this is not the case for UC, likely speaking to the paucity of data, mainly case reports and series, regarding this issue in adults. Resultantly, strong prevalence data in adults regarding involvement of the stomach, duodenum, and esophagus are lacking, with the latter two being even more poorly characterized. For instance, one case series and literature review detailed the rarity of esophageal presentations, which were typically dysphagia, not uncommonly with complications such as stricturing (>33%), and importantly manifesting as the initial diagnosis of CD in 55% of cases identified [6]. Interestingly, in a large recent study of 898 pediatric IBD patients, The UC cohort had 4% with upper GI involvement, in particular 0.8% with involvement of the esophagus or duodenum. Moreover, 5% of the UC cohort had no rectal involvement. The CD cohort had 22% of patients with upper GI involvement [7].

In recent years, there has been a larger concern for the prevalence and significance of upper GI disease in adult IBD patients and in particular the entity of gastroduodenitis associated with UC (GDUC) [8]. One such case series in adults suggested a significant relation between both pan-colitis and low dose prednisolone with GDUC; the former reflects disease severity and the latter reflects immunosuppressive effect. The authors also commented on the strong tendency of these patients to have undergone proctocolectomy with ileal pouch anal anastomosis (IPAA) with development of pouchitis prior to GDUC presentation. None of these patients had CD-like manifestations in their esophagus [8].

There are also cases of sequential or* de novo* CD, with presentations that can occur proximal to the original IBD colonic lesion, but in the majority of cases it tends to be in the pouch, that is, a “Crohn's pouchitis” after the IPAA. In cases such as these, CD transformation is thought to arise from both an altered microbiota and surgical-induced stress state in the setting of a genetic susceptibility [9].

This case also highlights consideration for the questionable entity of coexistent CD and UC. A search of the literature yields only a handful of older case reports, one review by Chen* et al.* noted the following: ages ranging from 20 s to 30 s, a fairly equal gender distribution, and an equal distribution of initial CD with addition of UC and* vice versa*. In the reported cases, CD manifested commonly as ileocolonic disease and it was never proximal to the jejunum. In the cases with UC as the original manifestation, it was never pan-colitis [10]. Our patient is distinct in having UC pancolitic features with distinct esophageal involvement. As noted above, esophageal involvement is rare, and when it does occur, it is commonly the presenting symptom [7]. It is acknowledged that former diagnoses of coexistent disease may have simply reflected the limits of historical testing and that sharper diagnostic instruments would reveal many of these reported cases, or even IBDU cases, to be atypical CD processes.

In conclusion, this case report details a patient with long-standing pan-UC who in the setting of new upper GI complaints displayed findings suggestive of an esophagogastroduodenal CD-like process. While the data is not conclusive, the evolving presentation of this case raises legitimate clinical issues. Firstly, a case such as this may trigger clinicians to more aggressively work up new upper GI symptoms in a presumably “well-controlled” UC adult patient that are otherwise thought to be benign in nature. Secondly, such findings would force the reconsideration of the initial UC diagnosis into either an atypical UC such as an entity similar to GDUC, a CD variant, or even IBDU. If a new designation were then decided upon, the consequence upon the patient, by utilization of differently targeted therapies, could be substantial.

## Figures and Tables

**Table 1:** IBD serologic panel.

Assay	Result (EU*/mL)	Reference (EU/mL)
ASCA IgA ELISA	7.3	<8.5
ASCA IgG ELISA	7.7	<17.8
Anti-OmpC IgA ELISA	8.6	<10.9
Anti-CBir1 IgG ELISA	33.4	<78.4
Anti-A4-Fla2 IgG ELISA	39.0	<44.8
Anti-FlaX IgG ELISA	39.2	<33.4
Autoantibody ELISA	52.3	<19.8
IFA perinuclear pattern	Detected	Not detected
DNase sensitivity	DNase sensitive	Not detected

*EU: equivalent units.

## References

[1] Baumgart DC, Sandborn WJ (2012). Crohn's disease. *The Lancet*.

[2] Ordás I, Eckmann L, Talamini M, Baumgart DC, Sandborn WJ (2012). Ulcerative colitis. *The Lancet*.

[3] Tremaine WJ (2012). Is indeterminate colitis determinable?. *Current Gastroenterology Reports*.

[4] Melmed GY, Elashoff R, Chen GC (2007). Predicting a change in diagnosis from ulcerative colitis to Crohn's disease : a nested, case-control study. *Clinical Gastroenterology and Hepatology*.

[5] Silverberg MS, Satsangi J, Ahmad T (2005). Toward an integrated clinical, molecular and serological classification of inflammatory bowel disease: report of a Working Party of the 2005 Montreal World Congress of Gastroenterology. *Canadian Journal of Gastroenterology*.

[6] Rudolph I, Goldstein F, DiMarino AJJ (2001). Crohn's disease of the esophagus: three cases and a literature review. *Canadian Journal of Gastroenterology*.

[7] Levine A, De Bie CI, Turner D (2013). Atypical disease phenotypes in pediatric ulcerative colitis: 5-year analyses of the EUROKIDS registry. *Inflammatory Bowel Diseases*.

[8] Hori K, Ikeuchi H, Nakano H (2008). Gastroduodenitis associated with ulcerative colitis. *Journal of Gastroenterology*.

[9] Li Y, Wu B, Shen B (2012). Diagnosis and differential diagnosis of Crohn's disease of the ileal pouch. *Current Gastroenterology Reports*.

[10] Chen GI, Saibil F, Morava-Protzner I (2002). Two for one: coexisting ulcerative colitis and Crohn's disease. *Canadian Journal of Gastroenterology*.

